# Low-dose PD-1 inhibitor combined with lenvatinib for preemptive treatment of recurrence after liver transplantation for hepatocellular carcinoma: Case report and literature review

**DOI:** 10.3389/fonc.2022.951303

**Published:** 2022-09-02

**Authors:** Xin Jin, Kangjun Zhang, Taishi Fang, Xinchen Zeng, Xu Yan, Jianxin Tang, Ziming Liang, Linjie Xie, Dong Zhao

**Affiliations:** Division of Liver Surgery and Organ Transplantation Center, Shenzhen Third People’s Hospital, Second Affiliated Hospital of Southern University of Science and Technology, National Clinical Research Center for Infectious Disease, Shenzhen, China

**Keywords:** AFP, hepatocellular carcinoma, immune checkpoint inhibitor, liver transplantation, tumor recurrence

## Abstract

Orthotopic liver transplantation (OLT), as one of the curative methods for the treatment of hepatocellular carcinoma (HCC), has brought hope to patients with HCC. However, treatment options for HCC recurrence and metastasis after liver transplantation are limited. Immune checkpoint inhibitor (ICI), such as programmed cell death protein 1 (PD-1) inhibitor, have been successfully used in advanced or metastatic HCC, but the data on the safety of PD-1 inhibitor after liver transplantation is limited. In this article, we report a 47-year-old patient with acute-on-chronic liver failure and multiple HCC who was successfully treated with liver transplantation. On the 45th day after OLT, the patient’s alpha fetoprotein (AFP) and lens culinaris agglutinin-reactive fraction of AFP (AFP-L3) were increased, and imaging examination showed no residual tumor. The patient had high risk factors for tumor recurrence before operation, so the possibility of tumor recurrence was considered. When the tumor markers showed an upward trend, we immediately treated the patient with lenvatinib 8 mg, after half a month, the AFP and AFP-L3 continued to increase compared with before. Then we used low-dose nivolumab 40mg, the patient’s AFP and AFP-L3 gradually decreased. One month later, a second low-dose nivolumab 40mg was given, and the patient’s tumor markers gradually decreased to normal. No acute rejection and other complications occurred during the treatment. So far, we have followed up this patient for 2 years, and no tumor recurrence was observed. To our knowledge, this is the first reported case using a low dose of nivolumab in combination with lenvatinib to prevent recurrence of HCC after liver transplantation.

## Introduction

The morbidity and mortality of hepatocellular carcinoma (HCC) remain high, and more than 700,000 HCC patients die worldwide each year, which brings a heavy burden to human society (1). At present, HCC is one of the main indications for liver transplantation. Milan standard is currently the most widely used international screening standard for liver transplantation recipients of HCC, but too strict criteria exclude many HCC patients from the transplant list. In recent years, the organ donation network system has been continuously improved. In order to benefit more patients with HCC, some extended criteria have been developed to expand the recipient range of liver transplantation for HCC, including the California University criteria, the Up-to-Seven criteria, and the Hangzhou criteria (2–4). Current research show that the 5-year recurrence and metastasis rate of HCC after liver transplantation is still as high as 30.6%. Some patients have tumor recurrence and metastasis within 1~2 years after liver transplantation, and the median overall survival after recurrence and metastasis is only about 1 year (5–7). The main risk factors for recurrence of HCC are poorly differentiated tumor, multiple tumors, microvascular invasion, and high preoperative alpha fetoprotein (AFP) and protein induced by vitamin K absence or antagonist-II (PIVKA-II) (8). The patient is in a state of immunosuppression after liver transplantation. Once tumor recurrence or metastasis occurs after liver transplantation, the disease will progress rapidly, which has become the main factor restricting the efficacy of liver transplantation (9).

For the recurrent tumors after liver transplantation, the effect of local treatment or systematic treatment is limited (10). Therefore, how to effectively control the tumor after operation and delay or even avoid its recurrence is important to improve the prognosis of HCC patients receiving liver transplantation. In recent years, with the in-depth research on immunotherapy, immune checkpoint inhibitor (ICI) represented by cytotoxic-T-lymphocyte associated antigen 4 (CTLA-4) inhibitor or programmed cell death protein 1 (PD-1)/programmed cell death protein ligand 1 (PD-L1) inhibitor has made great progress in the immunotherapy for advanced or metastatic HCC. However, its application in patients with tumor recurrence or metastasis after liver transplantation is still controversial. At present, there are some reports on evaluating the effect of ICI therapy after liver transplantation. Most clinical data show that immunotherapy has a poor prognosis in patients with recurrent metastasis after liver transplantation for HCC, mainly due to the induction of acute graft rejection and fatal liver failure after ICI at regular doses (11–14). But there are still some successful treatments (15–17). Recent studies have shown that the PD-1 receptor on the surface of peripheral T cells can be completely occupied when the dose of nivolumab is more than 0.3 mg/kg (18). Therefore, the use of lower dose of PD-1 inhibitor within the range of effective therapeutic doses may be able to maintain anti-tumor effect without increasing organ rejection. In the present study, we report a HCC patient with high risk factors for recurrence who successfully received low-dose PD-1 inhibitor for preemptive treatment of HCC recurrence after liver transplantation.

## Case presentation

**Figure 1:**
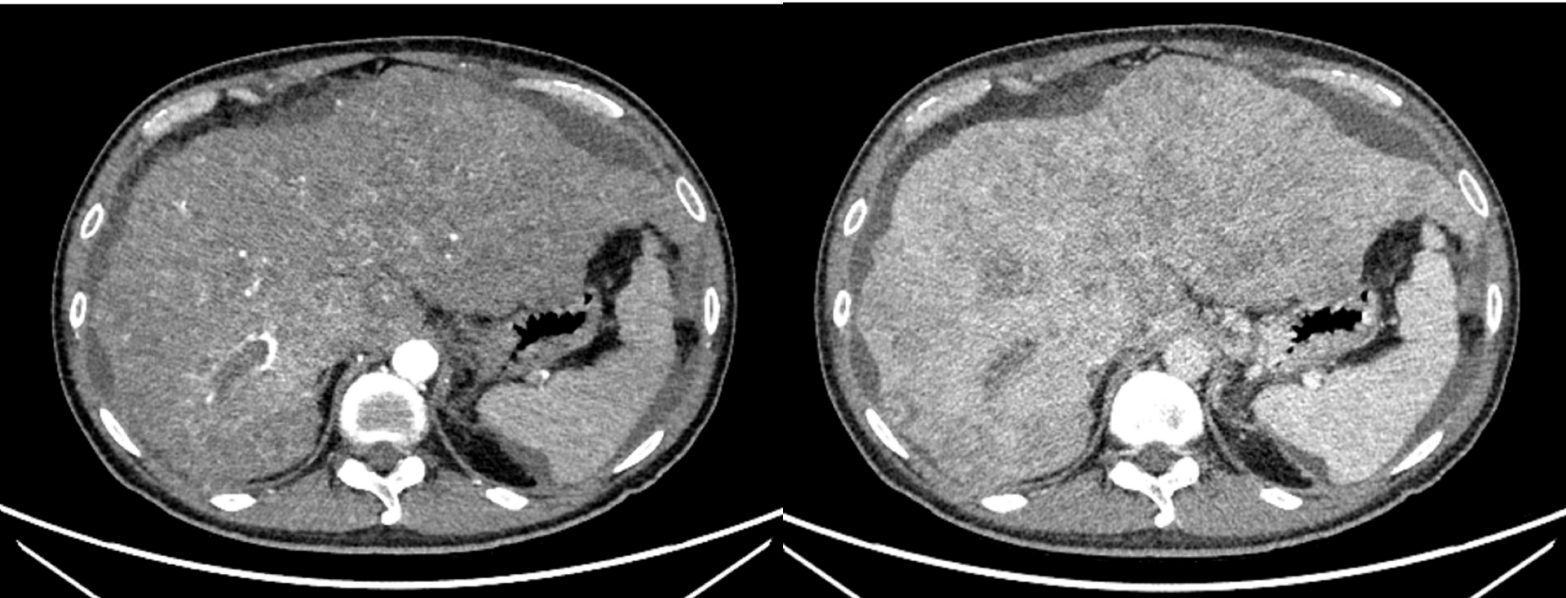
The preoperative CT images of the liver.

A 47-year-old male patient was diagnosed with acute-on-chronic liver failure, HCC, viral hepatitis B and decompensated cirrhosis in April 2020. Tumor markers showed that AFP exceeded 80000 ng/ml, lens culinaris agglutinin-reactive fraction of AFP (AFP-L3) was 133362.1 ng/ml, and PIVKA-II exceeded 30000 mAU/ml. Upper abdominal enhanced Computed Tomography (CT) showed HCC (number of lesions > 10), liver cirrhosis, and portal hypertension (**Figure 1**). Enhanced CT/Magnetic Resonance Imaging (MRI)/Positron Emission Tomography (PET) scan showed no extrahepatic or distant metastasis.

The patient received allogeneic orthotopic liver transplantation (OLT) in our hospital on May 20, 2020. Donor and recipient have the same blood type. Postoperative pathology showed diffuse multiple nodules, less than 1 mm from the liver capsule. Histopathology was moderately-poorly differentiated HCC. Immunohistochemical results showed PD-L1 (-), Happer-1 (+), Ki 67 (about 30%). The patient recovered smoothly after operation, AFP level decreased to 413.36 ng/ml, AFP-L3 level decreased to 110.7 ng/ml and PIVKA-II level decreased to 24 mAU/ml. One month after operation, he recovered and was discharged from the hospital.

**Figure 2:**
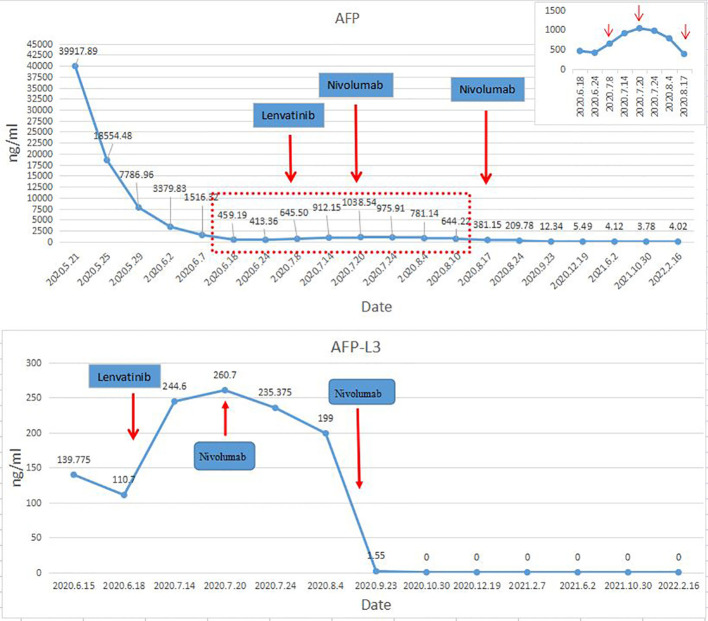
The trend chart of AFP and AFP-L3 after operation.

**Figure 3:**
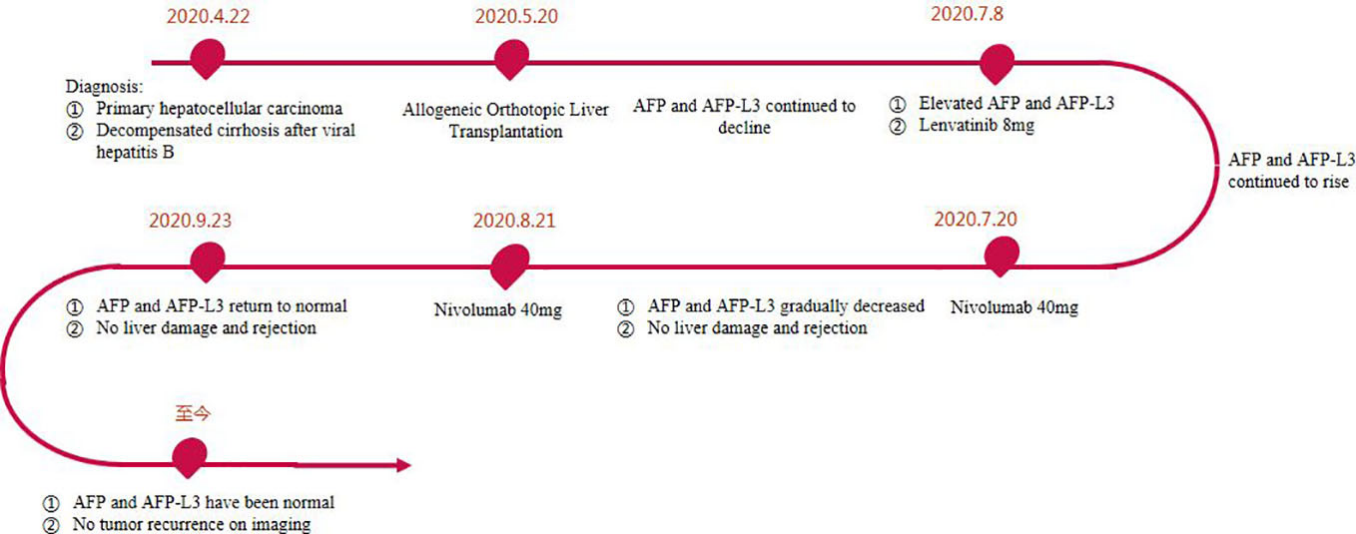
Treatment flow chart.

The postoperative immunosuppressive regimen was tacrolimus and Mycophenolate Mofetil. However, on the 45th day after OLT, it was found that AFP and AFP-L3 gradually increased, AFP increased from 413.36ng/ml to 645.5ng/ml, and AFP-L3 increased from 110.7ng/ml to 244.6ng/ml. But no tumor recurrence and metastasis were found on chest and abdomen CT. After comprehensive evaluation, lenvatinib 8mg/d was given. The immunosuppressant was adjusted to everolimus combined with Mycophenolate Mofetil (MMF). After 2 weeks of lenvatinib, AFP level increased to 1038 ng/ml, AFP-L3 level increased to 260.7 ng/ml, and PIVKA-II level increased slightly (**Figure 2**). In consideration of the patient’s risk of recurrence, and fully communicating the possible risks with the patient and his family members, we used a low-dose nivolumab 40 mg. On the 4th day after treatment, AFP level decreased to 975.91ng/ml, and AFP-L3 level decreased to 235.375ng/ml. It was evaluated as effective treatment. During this period, no rejection occurred, and the liver function was normal. After 15 days of treatment, the levels of AFP and AFP-L3 continued to decline. CT showed no tumor recurrence or metastasis. One month later, a second low-dose nivolumab 40 mg was given. The liver function is normal, and the levels of AFP and AFP-L3 continued to decrease and no rejection (**Figure 2**). At present, two years after liver transplantation, the levels of AFP and AFP-L3 have decreased to normal level. No tumor recurrence or metastasis was found by CT/MRI/PET-CT. The liver function is good and there is no rejection. The treatment flow chart is shown in **Figure 3**.

## Discussion

Tumor recurrence after liver transplantation seriously affects the long-term survival of patients. The recurrence or metastasis of HCC after liver transplantation is occult. With the progress of the disease, some recipients may have clinical manifestations such as anorexia, weight loss, fatigue, or pain at the site of bone metastases, or only show increased serum AFP and PIVKA-II levels (19). The peak period of postoperative tumor recurrence is within 2-3 years after operation. And recurrence occurring within 1 year after operation usually indicates a poor prognosis (20–21). Therefore, for recipients who exceed the original transplantation standards or have independent risk factors for tumor recurrence or metastasis, close monitoring should be performed. Early detection and active intervention of tumor recurrence or metastasis are essential to improve the long-term survival rate of liver transplantation recipients of HCC (22–23). For recurrent tumors after liver transplantation, the effect of chemotherapy is very limited. Although molecular targeted therapy drugs including sorafenib and lenvatinib have improved the efficacy of patients with recurrent HCC to a certain extent, they are still not ideal.

In recent years, with the development of immunotherapy, ICI has become a new first-line treatment for advanced HCC. The immune microenvironment of HCC is mainly composed of a variety of immune cells, extracellular matrix and cytokines, and nearly 25% of HCC patients are characterized by high infiltration of immune cells, expression of PD-1/PD-L1, active IFN-γ signal and lack of CTNNB1 mutation (24) and present a “hot tumor” state. Studies have shown that PD-1/PD-L1 is an important pathway for immune escape in the tumor immune microenvironment. PD-1 is a negative regulator of T cell activity. When PD-1 interacts with PD-L1 expressed by tumor cells, T cell activity is inhibited, thereby reducing the killing of tumor cells. Tumor growth will also accelerate the formation of new blood vessels, which in turn leads to the activation of multiple immunosuppressive pathways in the tumor microenvironment (TME) (25). Ma et al. (26) enrolled a total of 498 HCC patients in two cohorts. The results showed that high PD-L1 expression was positively correlated with high serum AFP levels, TNM stage and Barcelona clinic liver cancer (BCLC) stage. Calderaro et al. (27) found that tumor cells or inflammatory cells in the TME overexpress PD-L1 in 217 HCC patients, which can increase tumor invasiveness, manifested as high serum AFP levels, poor differentiation, and vascular infiltration. Many current studies have confirmed that targeting PD-1/PD-L1 can restore the scavenging effect of autologous immune cells on tumors, which is the theoretical basis for ICI therapy. Drugs targeting the above pathways have achieved significant curative effects in a variety of tumors including HCC (28–30). Current studies have shown that the objective response rate (ORR) of ICI monotherapy in HCC is 15% -20% (31–33), which is not satisfactory.

In view of the limited response rate of monotherapy, studies on immunotherapy in combination with other therapies are currently under way. The 2022 NCCN guidelines propose that atezolizumab combined with bevacizumab is the preferred treatment for first-line therapy. The IMbrave150 showed an ORR of 33% for atezolizumab combined with bevacizumab (30). As the results of clinical studies continue to be published, the treatment of lenvatinib combined with PD-1 inhibitor has also been greatly recognized. At present, several studies have confirmed the synergistic mechanism of lenvatinib combined with ICI (34–35). Yi et al. showed that lenvatinib can reduce the expression level of tumor PD-L1 and regulatory T cells differentiation by blocking FGFR4, thereby improving the efficacy of PD-1 inhibitor (36). The KEYNOTE-524/STUDY-116 showed an ORR of 46% for lenvatinib combined with pembrolizumab (37), while the STUDY-117 study showed an ORR of 76.7% for lenvatinib combined with nivolumab (38). Although there is a lack of data from large clinical studies of lenvatinib in combination with PD-1 inhibitor, these studies have confirmed the effectiveness of lenvatinib in combination with PD-1 inhibitor. For the immunotherapy of patients with recurrent HCC after liver transplantation, most of the current reports are case reports of the use of nivolumab or pembrolizumab, and there are no relevant guidelines for the treatment of such patients. Therefore, combined with the current treatment experience and the wishes of patients, we combined low-dose nivolumab with continuous use of lenvatinib as a treatment regimen.

ICI therapy has previously been considered inappropriate for patients after solid organ transplantation. Studies have shown that the incidence of acute rejection in patients with tumor recurrence after liver transplantation for HCC is about 35% (39). At present, the mechanism of transplantation rejection caused by ICI is still not fully clarified, but we consider that transplantation rejection is closely related to the imbalance of transplantation immune tolerance caused by ICI enhancing immune function. Both CTLA-4 and PD-1 play roles in transplantation tolerance. CTLA-4 is upregulated on the cell membrane and inhibits T cell function through a variety of mechanisms. The combination of PD-1 and PD-L1 can block PI3K/Akt/mTOR and Ras/MAPK/ERK signaling pathways, inhibit the differentiation of effector T cells, and lead to the incapacitation of CD8 + T cells and CD4 + T cells (40–41). The mechanism of rejection following PD-1/PD-L1 blockade is related to the activation of cellular immunity *via* the CD8+ effector cells, as well as the down-regulation of regulatory T cells. CTLA-4 blockade could promote the donor-specific T cell activation, Th1 polarization and protect alloreactive T cells from apoptotic death (42). Therefore, ICI not only activates the anti-tumor effect of T lymphocytes, but also easily causes the risk of acute rejection or loss of graft function of transplanted liver (42, 43). However, due to the suppressed immune function of patients after liver transplantation, the possibility of tumor recurrence or new tumor is increased. At present, there are some reports about ICI as an anti-tumor treatment after liver transplantation (**Table 1**), but most of them have poor prognosis and obvious adverse reactions. Gassmann et al. (12) reported that a patient with HCC recurred 2 years after OLT. Due to side effects and disease progression, the initial treatment of sorafenib was forced to stop. After comprehensive consideration, nivolumab 100mg was used for treatment. Abnormal liver function occurred after one course of treatment, and liver biopsy showed obvious rejection. After high-dose glucocorticoid shock combined with intensive tacrolimus immunosuppressive therapy, liver function continued to deteriorate, resulting in severe coagulation dysfunction. The patient eventually died of intracranial hemorrhage. Varkaris et al. (13) reported a patient with HCC complicated with liver cirrhosis recurred after liver transplantation. After using sorafenib, capecitabine and radiotherapy, the tumor progressed again after a period of stable disease. Further treatment with pembrolizumab, no obvious rejection occurred, but the tumor progressed rapidly. The recipient died 3 months later. Friend et al. (14) reported two patients with advanced HCC who relapsed, refractory, and progressive after OLT. Liver biopsy showed positive immunofluorescence staining of PD-L1. After receiving nivolumab, they all had irreversible acute rejection and finally died. We summarized the published case reports (**Table 1**), including 15 cases of tumor recurrence after liver transplantation. The incidence of acute rejection was 33%, and the mortality rate of patients with acute rejection was 26%, which was similar to the results of other reports.

**Table 1:** Clinical characteristics of immunotherapy in recipients with recurrence and metastasis after liver transplantation for hepatocellular carcinoma.

References	Sex	Age	Years after transplantation	Immune suppression	Checkpoint inhibitor / No.doses	Outcome	Rejection	Survival time (month)
Varkaris A (13)	Male	70	8	Tacrolimus	Pembrolizumab / 200mg	Progressive disease	No	3.0
Gassmann D (12)	Female	53	2	Everolimus +MMF	Nivolumab / 200mg	Organ failure	Yes	0.8
Friend BD (14)	Male	14	3	Tacrolimus	Nivolumab / N/A	Organ failure	Yes	2
	Male	20	4	Sirolimus	Nivolumab / N/A	Organ failure	Yes	1
Amjad W [16]	Female	62	1.3	Tacrolimus+MMF	Nivolumab / N/A	Complete remission	No	24^①^
Rammohan A (15)	Male	57	3.3	Tacrolimus+MMF+ Hormone	Pembrolizumab / 200mg	Complete remission	No	10^①^
De Toni EN (17)	Male	41	1	Tacrolimus	Nivolumab / 200mg	Progressive disease After Dissociated response	No	10
Al Jarroudi O (44)	Male	70	2.8	N/A	Nivolumab / 240mg	Progressive disease	Yes	4
	Female	62	1	Tacrolimus	Nivolumab / 240mg	Progressive disease	No	N/A
	Male	66	2	Tacrolimus	Nivolumab / N/A	Progressive disease	No	N/A
DeLeon T (45)	Male	56	2.7	Tacrolimus	Nivolumab / N/A	Progressive disease	No	1.2
	Male	55	7.8	Sirolimus+MMF	Nivolumab / N/A	Progressive disease	No	1.1
	Female	34	3.7	Tacrolimus	Nivolumab / N/A	Progressive disease	No	1.3
	Male	63	1.2	Tacrolimus	Nivolumab / N/A	Organ failure	No	0.3
	Male	68	1.1	Sirolimus	Nivolumab / N/A	Organ failure	Yes	0.9

^①^The recipients are still following up when the article is submitted.N/A, Not Applicable.

ICI, as a treatment to stimulate autoimmune function, obviously has great potential. Although most clinical data showed that the prognosis of patients with recurrent and metastatic HCC after liver transplantation was poor when immunotherapy was used, there were also successful cases in the real world. Rammohan et al. (15) reported a patient with lung metastasis after liver transplantation for HCC. The tumor progressed after sorafenib treatment, and the effect of pembrolizumab was obvious. Up to 10 months of follow-up, the patient continued to use pembrolizumab and sorafenib, which was well tolerated and had no radiological evidence of tumor recurrence. Amjad et al. (16) reported a patient with HCC complicated by viral hepatitis C cirrhosis. One year after liver transplantation, the tumor recurred with multiple metastases. Immunohistochemistry showed positive PD-L1 staining. Nivolumab was used after comprehensive consideration. After 6 months of treatment, liver lesions and all metastatic lesions were necrotic or disappeared, and the effect was remarkable. As of the publication of the report (24 months after the diagnosis of disseminated HCC), MRI or PET had not found evidence of HCC recurrence. This case is also the case with the longest survival time among the patients with recurrence of HCC after liver transplantation treated with ICI. In 2017, De Toni et al. (17) reported a case of HCC after liver transplantation. After tumor recurrence, the dosage of immunosuppressive drugs was adjusted, and then nivolumab was used. The patient has survived for more than 10 months, and no rejection reaction has been observed. Although the above cases all suggest the successful application of ICI in the field of liver transplantation for HCC, we believe that in the application of immunotherapy in patients with liver transplantation for HCC, it is necessary to precisely regulate the state of transplantation immune tolerance in order to maximize the effect of ICI and avoid the occurrence of acute rejection.

Therefore, in-depth study of the immune microenvironment of HCC and tumor recurrence after liver transplantation, combined with the specific conditions of patients to take individualized comprehensive treatment is the focus of attention at present. Numerous studies have demonstrated that the sequential implementation of different types of ICI, strength of immunosuppressive agents, time from transplantation to therapy, and immunogenicity of the particular organ grafted are factors potentially affecting allograft rejection risk and treatment responses (43, 46). PD-L1-positive patients have been reported to be at greater risk of acute rejection when receiving ICI (47). The negative expression of PD-L1 in our case may also be the reason for the small risk of acute rejection. In addition, choosing an appropriate dose of ICIs is also an important method to avoid rejection. Recent studies have shown that the PD-1 receptor on the surface of peripheral T cells can be fully occupied when the dose of nivolumab is more than 0.3 mg/kg (18). In studies of non-small cell lung cancer, reduction of nivolumab dose did not significantly shorten overall survival (48). Therefore, we believe that within the effective therapeutic dose range, the use of lower dose of PD-1 inhibitor may maintain anti-tumor effect without increasing the rejection of transplanted organs. In the case we reported, during the regular monitoring after liver transplantation, the levels of AFP and AFP-L3 were gradually increased, and PIVKA-II level was slightly increased, which was highly predictive of recurrence. Therefore, in the absence of imaging evidence of recurrence and metastasis, we used low-dose nivolumab combined with targeted therapy in advance, which showed a good prognosis. As far as we know, there is no example of preemptive use of low-dose ICI drugs in the treatment of tumor recurrence after liver transplantation, and the treatment process of this case has guiding significance for our future clinical work.

## Conclusion

ICI treatment after liver transplantation has a good effect only in some patients, and its application may cause fatal rejection, which is the reason why ICI treatment after liver transplantation is still widely controversial. In liver transplantation patients, there is still a lack of effective indicators that can effectively predict the prognosis of ICI treatment, so it is difficult to effectively select patients who may benefit from ICI treatment. The high risk of ICI in liver transplantation patients also limits the application of ICI in the treatment of recurrent tumors after transplantation. According to the current results, ICI treatment can be used for patients with recurrent tumors after liver transplantation is still lacking in complete clinical research. It needs to be further confirmed. However, a successful case of low-dose PD-1 inhibitor in combination with lenvatinib may open up a new option for the prevention of tumor recurrence after liver transplantation.

## Data availability statement

The datasets presented in this study can be found in online repositories. The names of the repository/repositories and accession number(s) can be found in the article/supplementary material.

## Ethics statement

Written informed consent was obtained from the individual(s) for the publication of any potentially identifiable images or data included in this article.

## Author contributions

All authors contributed to data analysis, drafting or revising the article, gave final approval of the version to be published, and agree to be accountable for all aspects of the work.

## Funding

This work was supported by Shenzhen Key Medical Discipline Construction Fund (SZXK079).

## Conflict of interest

The authors declare that the research was conducted in the absence of any commercial or financial relationships that could be construed as a potential conflict of interest.

## Publisher’s note

All claims expressed in this article are solely those of the authors and do not necessarily represent those of their affiliated organizations, or those of the publisher, the editors and the reviewers. Any product that may be evaluated in this article, or claim that may be made by its manufacturer, is not guaranteed or endorsed by the publisher.
